# Medical Progress, Local and General

**Published:** 1891-12

**Authors:** F. R. Cross

**Affiliations:** Ophthalmic Surgeon to the Bristol Royal Infirmary; Surgeon to the Bristol Eye Hospital.


THE BRISTOL

Medico-Chirurgical Journal.

DECEMBER, 1891.

### MEDICAL PROGRESS, LOCAL AND GENERAL.

The Presidential Address, delivered on October 14th, 1891, at the opening of the 18th Session of the Bristol Medico-Chirurgical Society.

BY

F. R. Cross, M.B. Lond., F.R.C.S. Eng.;

*Ophthalmic Surgeon to the Bristol Royal Infirmary;*
*Surgeon to the Bristol Eye Hospital.*

My first duty and pleasure in taking this chair is to express my thanks to you, the members of the Bristol Medico-Chirurgical Society, for the kind honour you have paid me in selecting me as your President.

Our Society, under its present constitution, was founded on February 24th, 1874, by forty-four members of the medical profession in Bristol and Clifton, "for the cultivation of medicine and surgery in all their branches." It was the resuscitation of an older Society under the same name, in which excellent work had been done until within a short period of the date mentioned.

Seventeen and a half years is not a very long time, and yet within it many of those who laid the foundations of our present satisfactory position have passed away. No less than four of my predecessors in this chair have gone to their last resting-place, leaving their work and example to us. The ready eloquence and mental acumen of Frederick Brittan, whose loss we have so recently had to deplore, made him an admirable first President; and his inaugural address on "Contagion and the Germ Theory" showed a kind of prescience of the extreme importance that this subject was about to bear in the development of medical knowledge. The second address from a President was given four years later by Henry Edward Fripp, upon a very similar subject, "The Doctrine of Contagium Vivum, and its Relations to Parasitic Disease." And since this time an address has formed an important unit in the duties of each succeeding President—a responsibility that must be borne, no matter how incompetent one may feel to undertake it.

A suggestion was made by Dr. Fripp, in the course of his address, that an occasional volume of original papers should be issued under the Society's auspices. The outcome of this was the publication in 1878 of the first volume of the *Transactions* of the Bristol Medico-Chirurgical Society, containing selections from the proceedings during the years 1874 to 1878. To this volume Dr. Fripp wrote a preface, characterised by much philosophic thought. Regarding the new departure, he said: "However unpretending as a literary effort, the publication of *Transactions* by a society marks its growing self-consciousness of present active power, and its faith in the present and prospective usefulness of such an undertaking. The self-criticism which it excites is also in itself a source of strength, and a powerful motive to further exertion. We may assume that local interests, which have slumbered, will again spring up in connection with the local volume of Medico-Chirurgical *Transactions*. Such a volume might even be welcomed as a proof that the medical centre of the West of England maintains its claim to be considered a teaching power, fairly abreast with the general progress of science, and able to sustain its old repute."

Within a year of resigning the chair to his successor, Henry Fripp was no more; and Crosby Leonard (who, although never President of the Society, was chairman of the meeting at which it was founded) had died a few months before him. The successor was one of very high professional experience, possessed of unusual surgical insight and ability, William Michell Clarke, who left us still in the prime of his intellectual vigour. Samuel Martyn, scientific physician and physiologist, had died while he was still President of the Society in 1876; so that of our first six Presidents only two are left—and may they long remain our surgical Nestors—Coe and Augustin Prichard.

In 1882 the second volume of *Transactions* should have been issued, but it seemed to several of our members inexpedient to publish papers so long after they had been read, and when time had deprived them of much of their original value; and, moreover, it was thought that a more comprehensive form of publication should be issued at shorter intervals.

Mr. Griffiths and Mr. Greig Smith proposed in committee "that a journal should be issued, to embrace the work of the medical men in Bristol and its neighbourhood," but they received little or no support from their colleagues on the committee. Shortly afterwards, however, the following resolution, which expressed a feeling that had become wide-spread amongst the members of the Society, was carried at a general meeting without any dissentient vote:—

"That, in the opinion of the Society, the time has come when the various medical and surgical interests of the West of England ought to be united by the publication of a periodical journal, which might appear under the auspices of the Bristol Medico-Chirurgical Society."

A sub-committee of nine members was appointed to make inquiries, and report thereon. It was afterwards decided, on the recommendation of this sub-committee, that the *Bristol Medico-Chirurgical Journal* should be published forthwith in half-yearly numbers, with Mr. J. Greig Smith as editor, and Mr. L. M. Griffiths as publication secretary. The detailed arrangements respecting the publication were left to the General Committee of the Medico-Chirurgical Society. A letter sent by the editor to the medical men of the neighbourhood contains passages which well express the reason for the appearance of the new *Journal*:—"When (other) provincial centres are each represented by vigorous and influential periodicals, it seems natural that a district, so large and populous as the West of England and South Wales, should also have its professional mouthpiece. Such a publication, issued in our midst, and supported by ourselves, would not only rescue from oblivion much that might otherwise be lost, but would also stimulate us more keenly in our efforts to advance medical science. The proposed *Journal* is not intended to rival existing periodicals, but to supplement them. Much valuable material is crowded out of our weekly journals for want of room, and a knowledge of this fact frequently discourages men from seeking to record cases and observations of great value. That there is room for the *Journal* there can be no doubt; its success depends entirely on the heartiness with which the medical men of the district will support it and write for it. The fact that the members of the Bristol Medico-Chirurgical Society are prepared to be its sponsors and guarantors is sufficient assurance that it will be broad and liberal in its views and aims."

Nothing could exceed the devotion and energy displayed by the editor and his colleague in the early conduct of the *Journal*. Between them was a rare combination of literary ability both for medical and for general subjects, good business capacity, and a full intention that the *Journal* should succeed. In Mr. Arrowsmith they found a publisher who, by his experience and readiness to advise, has given invaluable assistance.

After two half-yearly issues, the *Journal* appeared quarterly, as it does at present. Each year has shown a profit on the *Journal* account, and this has been allowed to collect, until an aggregate sum of £260 has been reached. The *Journal* has never been any charge upon the Society, and has been delivered without any cost to the members.

In connection with the life of this *Journal* was accumulating a large amount of medical literature, which our editors had carefully stored, and which they intended, when the time came, to make the nucleus of a thoroughly good medical library.

Mr. Griffiths, when he was President of the Society, proposed that this project should be carried out; but no definite step was taken in this direction until the committee, acting upon a request from a large number of members of the Society, presented a report at the annual meeting last year, in which the details for the formation of the library of the Bristol Medico-Chirurgical Society were set out.

The library was formally opened, at 28 Berkeley Square, by Mr. S. H. Swayne, on January 5th, 1891.

I will venture here, as this is the first annual meeting of the Society that has taken place since that date, to express our thanks to Mr. Greig Smith, who, as editor of the *Journal*, laid the foundations of the library, and by his unceasing advocacy has prepared the way and assisted in its development; and to Mr. Griffiths, upon whom, as our first librarian, the labour of completion of the work has entirely fallen, and to whom also is due the credit of the financial position of the *Journal*, which has allowed the liberal help of £100 towards the foundation of the library.

There is an unusually complete set of about eighty journals lying on our tables. The books number 1,800, and include many of the classic works on medicine. We have to thank private liberality for the greater number of these, our most beneficent donor being the British Medical Association. No more important step has ever been taken in the medical life of Bristol than the foundation of this library, and in its satisfactory completion and continuance we may all assist. The recent September issue contains a list of the books we at present possess, and your attention is particularly drawn to the Transactions and Reports marked with an asterisk because they are incomplete.

It is just a quarter of a century since I entered as a student at King's College, London. Anatomy was as well taught then as it is now, but Histology was in its infancy, and Physiology, as at present understood, was a sealed book. Very little was known of the workings of the nervous system. Hughlings Jackson was busy in collecting clinical and pathological facts, but Ferrier's work had not been conceived.

Pyæmia, erysipelas, and hectic fever were constantly present in our hospital wards; but Lister was just beginning to grasp the importance to surgery of what Pasteur was doing in another field of work, and the remarkable effects which had followed the use of carbolic acid on the sewage of the town of Carlisle induced him to try what result this remedy would produce on wounds.

In 1867 his earlier papers on "Antiseptics" were published, showing that perfect healing occurred under a solid crust of blood and carbolic acid, the absorbing power of granulations on the exfoliation of bone, and the value of carbolic paste (with linseed oil and carbonate of lime) as an antiseptic dressing. Torsion and acupressure of vessels—in the place of the old ligature, which used to ulcerate right through the artery, and was a prolific cause of secondary hemorrhage—was just being advocated, but was soon superseded by the knowledge that the ligature under antiseptic treatment might be cut short and allowed to absorb. Yet surgeons would not recognise these things, although attention was further drawn to them by Syme, to whom it was evident that an immense improvement had taken place in surgical practice, which promised to produce a great diminution in human suffering and danger.

At the same time Thompson and others were busy in diseases of the urinary tract. Lithotrity was on its trial, and Clover was suggesting the importance of washing out the *débris*. Spencer Wells had just written his first edition of *Diseases of the Ovaries: their Diagnosis and Treatment.*

The separate entity of typhus and typhoid fever had lately been admitted by all the best authorities in England, but this position was still assailed by many writers.

In 1867, also, Cohnheim's paper on the "Emigration of the Blood Corpuscles" was published, and the true pathology of inflammation illustrated and explained.

Lockhart Clarke's investigations on the histology of locomotor ataxy, an increasing confidence in the use of nitrous oxide for anæsthesia, and the introduction of Politzer's bag for injection of air into the Eustachian tube, while this is opened by the action of the tensor palati in swallowing, are other indications of the position of medical knowledge at this time; and it was not until 1872 that hyperpyrexia in fevers was combated by the application of cold.

Not long ago we were content with a knowledge of pathological changes in the tissues, and their effects upon the organ and the individual. Modern bacteriological research aims at a recognition of the way in which these abnormal tissue-changes are brought about, and seeks to prevent the development of the earlier causes, and to destroy them before they have damaged the body. The triumphs of surgery seem to have no bounds, and modern diagnosis is only delayed by the limits of physiology.

Yet, despite the claims that we really possess to a sound scientific knowledge of the processes of life, death, and disease, I think we appeal but little to popular appreciation. The public cannot understand the foundations in biology upon which our art is based, and they entirely misjudge the possibilities of our judgment or dexterity. The poor give credit only to the drug prescribed; the wealthy, whose experience is in change of fashion rather than in gradual progress, encourage the development of every kind of new craze in professional labelling or sectarianism.

In such a Society as ours, truth should be sought for as well as originality. We want to make the ground sure as we proceed; and for this purpose it is essential that all who really have experience should give it us. There is much yet to be learned about the etiology and inheritance of disease. Gout, syphilis, struma, alcoholism—for instance—can only be perfectly handled by those practitioners who have a knowledge of its working through several generations of a family.

Points concerning the life history of disease cannot be satisfactorily ascertained in hospital wards, partly from want of time, mainly because the witnesses are illiterate and untrustworthy. Private practice also is the best field for accurate testing of the value of remedies; and the general practitioner, by his breadth of knowledge, should always be a welcome critic on any branch of specialism.

Just at the commencement of my student days strong controversy was waging against specialism, and the formation of special-hospitals, which had been forced into existence by the short-sighted policy adopted by the larger general institutions, whose managers declined to recognise the advisability of any subdivision of labour in medical work, beyond that of medicine from surgery, except perhaps diseases of women and of the eye.

The consequence of this policy was that many comparatively obscure or neglected branches of the healing art had fallen into the hands of quacks, and were only rescued from them by orthodox practitioners, who started here and there small institutions for the treatment of some particular organ. Concentrated study led to increasingly successful treatment, attendance of patients and pecuniary support both increased; and when students found fresh fields of study, which their own school of medicine did not afford, and, young men, such as Jonathan Hutchinson and Morell Mackenzie, accepted posts upon the staffs, it was evident that something was wanting in the opportunities afforded at the general hospitals.

Both the *Lancet* and *British Medical Journal* strongly advised the formation of special departments in the hospitals, to which medical schools were attached, both for the sake of the students, and to prevent the quite unnecessary competition in charitable medical relief, which was rapidly developing, and which ought to have been prevented in anticipation by an earlier reform.

It was felt that the large general hospitals should be as perfectly equipped as possible, to deal with disease in all its phases; and that they should afford complete instruction to all classes of practitioners.

The principle of special departments was soon accepted; some few pure specialists were appointed, but in most cases the work was divided amongst certain existing members of the hospital staffs. Thus, at King's College, Dr. George Johnson took charge of the throat, and Dr. Duffin of diseases of the skin; while at St. Bartholomew's, eye, ear, skin, and orthopædic departments were founded; and at St. Mary's, one for diseases of the throat.

Thus patients became classified to their own advantage, for the better advance of science, and for the convenience of both teacher and learners. There exists now in almost all the hospitals, both in London and the provinces, to which medical schools are attached, special departments for diseases of women, the eye, throat, ear, and skin. Each one of them (and the same may be said of dentistry) is but a branch of the tree of medicine,—not a separate study of itself, but one that can only be properly understood and dealt with through a competent appreciation of its associates and a good knowledge of the general principles that regulate the whole science.

Medicine cannot be split off into small sections, for there is overlapping at every point; yet the field of work is so vast that there is surely room for division of labour; and the history of the progress of our art shows that the greatest advances have been made by those whose attention has been mainly directed to one particular subject.

In this country most of the best original work has been done by general surgeons and physicians, and for this reason some have been apt to question somewhat the advantage of the existence of specialists. It is obvious, however, that the progress has been made by individual concentration of thought in one direction, and a comparative lack of interest in others. The labour has been distinctly specialised, though the workers would refuse the designation "specialist." The *prestige* of the great hospitals and medical schools has naturally kept about them the best young intellects in the profession, particularly those who could afford to study, and wait in patience for the tide to fortune; and when these have been elected to hospital posts, they have been well content to settle happily in the midst of the conditions they have found about them, and there has been little tendency to encourage changes.

It will be admitted that, among the best known names of the younger members of eminence in the profession, many are prominent in association with some particular branch of work, and their consultation practice will mainly lie in the direction thus indicated. It is interesting to note how often the winner of the Jacksonian Prize Essay has come to be the leading authority on the subject of his paper.

The fact of gaining the prize was an early indication of special aptitude, or of opportunity for dealing with the problems involved in the inquiry. Other men of ability, for various reasons, have become associated with the special hospitals in London, and have been most assiduous penmen, the numerous journals now devoted to each special branch being both the cause and effect of this literary energy. The interchange of foreign thought and practice through these channels has led to very rapid development of knowledge amongst the workers in every department of medicine and surgery; and whilst the German in his laboratory gets credit for minuteness of detail and much originality of suggestion, the English can lay claim, through the large number of patients attending their clinics, to unusual opportunities for practical experience, and for great aptitude in the treatment of disease.

Much excellent work has been done by our specialists, who are, I believe, per head of the population, in a very small minority compared with those of any other country. In Paris about two in five practise almost exclusively some speciality, and a large proportion are found in every good-sized town in Germany.

Exclusive specialism may easily be carried in small departments beyond legitimate bounds; its practice no doubt tends to narrowness of view, and has a temptation to over-estimate its own importance. Still, it gives opportunity for refinement in examination of minute particulars, as well as wide observations from which comes skill in diagnosis and operations; and even if the importance of a symptom in the organ complained of is over estimated and too much attention is paid to its remedy by local means, the mistake is no greater than is frequently repeated by those who see in everything a constitutional state, and who, without taking the trouble to satisfy themselves thoroughly as to the local conditions, prognose recovery from the local malady as the health gets better or as the weather improves. On the other hand, many practitioners are well informed in most branches of special work, and use with complete mastery many of the instruments of precision which have become so numerous and so essential. The organ at fault requires the first intelligent consideration; and it is only after a competent examination of local defect that the constitution should be blamed and physicked.

At the same time, however definite the local lesion and however special the complaint, the fullest consideration must of course be given to the constitutional state.

All true knowledge tends towards simplicity; and as the essential principles which regulate the treatment of the various pathological states, wherever they occur, become more definite, and the anatomical peculiarities of each organ are more thoroughly appreciated, and the various methods and means for their thorough examination become more generally understood, much of the work that is now the province of the specialist will become the property of the family physician; whilst there always remain intricacies in diagnosis, refinements and difficulties in operative treatment, and cases of unusual occurrence, for which the aid of the specialist will continue to be sought. Moreover the element of time must be considered, and the doctor who is engaged in a single class of work will more rapidly and thoroughly form a good opinion in a case that is an ordinary one to him, than will his colleague who has equal ability but a wider range of subjects on which to expend his energy.

One result of this great development of medical energy, in association with the charitable impulses of our time, has been the present growth of charitable medical relief. Cottage hospitals are springing up everywhere; first-rate operators and diagnosticians are found in every county infirmary. In London there are 8,000 beds, which annually relieve about 75,000 in-patients. One million one hundred thousand out-patients is an enormous percentage on a population of five millions; and thirteen hundred honorary medical officers give their services for the skilled help required by this mass of invalids.

The recent inquiry by the Committee of the House of Lords shows that, on the whole, the great voluntary hospitals of London are well administered by their honorary committees and medical staffs, and compare most favourably with the management of the rate-supported hospitals. Still, it is evident that charitable medical relief in London and elsewhere requires organisation, in the interests of philanthropy, of the charitable public, of the medical profession, and of the patients themselves.

First, it is evident that in London the hospitals are too irregularly distributed. Thus, within a mile radius of the Middlesex are 8 general and 26 special hospitals, providing a total of 2,050 beds; in addition to dispensaries and poor-law infirmaries. In other districts there is scarcely any charitable medical relief at all. In many of the well-to-do suburbs, hospitals seem to be springing up where probably they are not required, and, by attending patients who could well afford moderate fees, are interfering with the local junior practitioners.

It would be clearly advantageous if some central authority existed, which could decide before any new hospital is started whether there is a necessity for its existence in the common good. If new hospitals are to be built, that which seems to be particularly needful, specially in the neighbourhood of London and all large centres, is the establishment of institutions for convalescents, to which (after serious illness, and long before they are able under present conditions to leave the hospital ward) patients might be sent. The fundamental work of the hospitals consists in the treatment of in-patients, and by far the largest part of their expenditure is devoted to this object.

But little can be said against *in-patient* hospital aid. It would be difficult to refuse the hospital bed to anyone seriously ill; yet as hospitals were originally founded by the rich for the benefit of the poor, and as medical men (with the exception of the juniors holding the resident posts) willingly and rightly give the best of their abilities quite gratuitously, inquiry should be made concerning the recipients of the help, and those who can afford to do so should be obliged to pay.

Nowadays the position of the working-classes has immensely improved, and they subscribe very liberally to the hospital expenses. The Birmingham Saturday fund, in 1891, handed over £10,300 to be divided amongst the medical charities as a free gift, without any specified returns in *quid pro quo;* and besides this sum, they contributed £7,000 to the hospitals in return for tickets. The artisan classes in the principal provincial towns contribute nearly £60,000 a year, at a cost on collection of 5 per cent. (as against £16,000 subscribed in London, at a cost of 10 per cent.). In Bristol, for each of the past three years, the Hospital Sunday fund has collected about £600, which was paid over to the charities in full, without any charge whatever for collecting; and the Hospital Saturday fund at the same period amounted to an average of about £1,200. There seems some reason for believing that as the money subscribed by the working-classes increases, that from the middle and upper classes gets less; and among those who fail to subscribe are small employers of labour. The amount received from subscriptions, especially to the general hospitals of London, seems to be relatively small. Legacies are the most fruitful source of revenue, representing about 20 per cent., but varying much in amount, as they are mainly left to the larger London and provincial hospitals, where the most difficult cases are sent for treatment: this fact is remembered by the wealthy when arranging to leave behind them a tithe of their wealth in the best interests of humanity. Hospital committees are importunate in their appeals for aid, and yet how many of them barely pay their way! Indeed, few hospitals could continue to exist without the help of legacies, which ought properly to be invested, and not to be used for paying debts.

Every unfair use of the charity, therefore, should be by them resented as an expense which they cannot afford. Moreover, as the doctors are giving services which if charged for on the most moderate scale would involve an enormous expense to the community, the representatives of the subscribers should undertake to see that no patients receive charitable medical relief who can afford, under the circumstances, to pay for the services of their ordinary medical man. No member of the profession would wish to receive fees from those who are really unable to pay them, and we are always anxious to be of service to any sufferer from illness; but it is the duty of relatives and friends to help, even if they have to deny something to themselves, rather than to take advantage of the ease with which unpaid medical assistance can be procured.

"Do as you would be done by" should help the sick and deserving; but the recipient and his friends should remember the motto as well as medical charity. Neither the hospitals nor the medical profession can afford to give away their stock-in-trade; and it is clearly unjust that they should be asked to do so by those who can well afford to pay for it. Good advice can be obtained at the present day at a very moderate cost, in comfort, and without the loss of time that charitable relief usually involves. The definition of a proper applicant for out-patient hospital relief is not an easy one. Any urgent case should receive immediate treatment; but after this "First aid," the patient's claim upon further charitable help must depend upon whether he can fairly be asked to pay for such medical assistance as the nature of the case requires. It is of course most difficult to draw a line between the well-to-do, who ought to pay a private doctor, and the *industrious poor*. The *destitute poor*, who require food, fire, and clothing, rather than medicine, should be treated through the agency of the Poor Law. A resolute effort has been made in Birmingham (which may fairly be considered the pioneer in perfecting the systems of the collection and distribution of workmen's contributions) to grapple with

19

the difficulties concerned in the proper application of medical charity.

The Report of the Birmingham Hospital Reform Inquiry Committee, consisting of representatives of the medical charities of the city and other organisations allied to them and of representatives of the medical profession, is well worthy of study. It is published by Osborne & Son, 84 New Street, Birmingham.

Without relying absolutely on statistics, it is evident that in most large cities the mass of patients attending the hospital out-patient rooms is more than can be satisfactorily dealt with. Exclusion of a considerable number of them is to be recommended from two aspects: some of the applicants are too well off, while others are not sufficiently ill.

The Provident Branch of the Metropolitan Hospital, in Kingsland Road, was founded to try and prevent locally the abuse of out-patient departments. The work was limited to people of the district, within a mile radius of the Dispensary, who were unable to pay medical men. A wage limit was fixed of 21/- for a bachelor, and of 35/- for the whole earnings of a family. At University College there is, I believe, a wage limit of 30/- for out-patient relief. But a paid income test is quite inadequate. It falls unfairly upon a man with a sickly wife or children, who is thus put to constant expense on their behalf; or upon one whose family are suffering from sudden severe illness. A complicated malady or an operation renders hospital aid justifiable to some persons who would have no right to come for an ordinary ailment.

The hospitals should decline trivial cases, for which sick clubs and provident dispensaries afford medical aid. An impulse would thus be given to self-help amongst the labouring classes, the friendly societies would increase and prosper, and an additional income would reach the medical men associated with them.

It may here be urged that the provident dispensary or club is open to the abuse that it may be used by those who ought to pay more liberal fees. Well, then the rules must be so framed as to prevent it, by a wage limit or some similar means. If medical men were united as to what is fair to their patients and just to one another, many of the difficulties in these questions would disappear, and fees would be maintained on an equitable basis. The overcrowding of out-patient departments with trivial or incurable cases keeps waiting those who are suffering from more serious maladies, and wastes the energies of the medical staff.

Large firms and bodies of workmen, I think, should employ private medical men at centres convenient for the workshops; and the hospital out-patient rooms should be opened for any cases sent on by medical men which require special help, or are of a more serious nature than they have time to attend to; or that require operations, or any kind of instrumental or mechanical treatment; or in which the artisans themselves require further advice.

The neighbouring practitioners would thus be relieved of any feeling as to unfair hospital competition; trivial cases would be excluded, and at the same time an increased guarantee would be afforded of the financial position of the patients. Time would be saved for all concerned. Statistics based on the quantity of patients would be replaced by those based upon quality of work, and students of medicine would have the advantage of being leisurely taught on selected cases, amply sufficient, both in number and in variety of kind and degree, for all the exigencies of future practice.

Any question that has reference to students is of particular interest just now to us in Bristol, for the medical wing of University College is rapidly approaching completion; and I am sure those members of the Medico-Chirurgical Society who, like myself, have no connection with the School of Medicine most heartily congratulate the faculty on this step towards its further prosperity.

A hospital is a great gainer by being affiliated to a medical school. Facts tend to show that those hospitals to which students are attached are the most popular with patients, who like full attention and inquiry to be given to their cases. The students stimulate the efforts and interest of their teachers towards a high standard of medical and surgical work. They give much help in working out the detail and history of cases, in making chemical and microscopical examinations, and in assisting generally in the work of the wards, particularly as surgical dressers. They save time to the staff, and thus relieve them for a higher concentration upon the more abstruse and difficult cases. Were it not for the medical school, paid officers would have to replace the clerks and dressers in many of the larger hospitals.

Some of our fellow-citizens in Bristol have shown they understand that there are advantages in the possession of a school for medicine in their midst, for they have given considerable help towards the erection of the new school buildings. The subscribers would have been much more numerous if the true facts as to these advantages were better understood. Moreover, our Medical School faculty has earned the gratitude of those who are concerned or interested in the development of higher scientific training; for it was from the old Medical School, in Park Row, that the proposition originally emanated to start in Bristol a united College for science and literature. University College is the outcome of this conception; but circumstances have rendered the union of the College and of the Medical School less complete than, for their mutual advantages, it ought to be. The completion of the new wing will, I hope, culminate in their incorporation and in their continued prosperity.

I believe that the Bristol Medical School is an advantage to every medical practitioner amongst us; and it is evident that it materially fosters and assists the work done by our medical associations.

Gentlemen, we must all continue to be students as long as we practise medicine. May we all work together in the good old friendly rivalry of former student days; and may our meetings in this Society always tend, not only to our increasing wisdom, but to our continuous friendship.

